# The Cambridge Cognitive and Psychiatric Assessment Kit (CamCOPS): A Secure Open-Source Client–Server System for Mobile Research and Clinical Data Capture

**DOI:** 10.3389/fpsyt.2021.578298

**Published:** 2021-11-17

**Authors:** Rudolf N. Cardinal, Martin Burchell

**Affiliations:** ^1^Department of Psychiatry, University of Cambridge, Cambridge, United Kingdom; ^2^Cambridgeshire and Peterborough NHS Foundation Trust, Liaison Psychiatry Service, Cambridge, United Kingdom

**Keywords:** clinical informatics, research data capture, cognitive assessment, psychology, psychiatry, clinical neurosciences, information governance

## Abstract

CamCOPS is a free, open-source client–server system for secure data capture in the domain of psychiatry, psychology, and the clinical neurosciences. The client is a cross-platform C++ application, suitable for mobile and offline (disconnected) use. It allows touchscreen data entry by subjects/patients, researchers/clinicians, or both together. It implements a large and extensible range of tasks, from simple questionnaires to complex animated tasks. The client uses encrypted data storage and sends data *via* an encrypted network connection to a CamCOPS server. Individual institutional users set up and run their own CamCOPS server, so no data is transferred outside the hosting institution's control. The server, written in Python, provides clinically oriented and research-oriented views of tasks, including the tracking of changes over time. It provides an audit trail, export facilities (such as to an institution's primary electronic health record system), and full structured data access subject to authorization. A single CamCOPS server can support multiple research/clinical groups, each having its own identity policy (e.g., fully identifiable for clinical use; de-identified/pseudonymised for research use). Intellectual property rules regarding third-party tasks vary and CamCOPS has several mechanisms to support compliance, including for tasks that may be permitted to some institutions but not others. CamCOPS supports task scheduling and home testing via a simplified user interface. We describe the software, report local information governance approvals within part of the UK National Health Service, and describe illustrative clinical and research uses.

## Introduction

There are strong potential advantages to the electronic capture of information relevant to cognitive and psychiatric assessment. Measurement-based care improves clinical outcomes (1). Some simple standardized scales are in widespread clinical use, such as for affective disorders or cognitive examination [e.g., (2, 3)], but if the information is captured using pen and paper then its subsequent clinical accessibility and/or availability for research is limited, and tasks must be scored by hand, taking time and introducing the potential for error. More complex computerized tasks are being translated from research to clinical use [e.g., (4)], but the clinical application of such animated tasks can be limited by practical considerations such as availability. Clinical and research assessments involve the documentation of a considerable quantity of information. Whether in a research or a clinical environment, and whether in an environment using paper-based or electronic health records (EHRs), there are incentives to capture such information electronically and in a standardized and structured fashion (5, 6). These incentives include a potential reduction in the effort of data capture; the ability to reproduce information accurately, legibly, and fast; the ability to appreciate trends over time; and the ability to analyse data for research or administrative purposes later. Information entered directly by patients can be used for screening and other purposes [e.g., (7)]. Rapid electronic systems can also capture information on outcomes that may not otherwise be measured routinely, such as quality of life indicators, used as the basis of many health economic measurements (8).

However, software for this purpose must overcome several potential pitfalls. First, for an application to enter widespread clinical use, it should save clinicians time, or at least place minimal time burden on clinicians. It should be quick to use and available at the bedside, in the clinic, or wherever a clinical or research encounter may take place. Second, users or institutions may be deterred from using software that is proprietary or closed-source (9–12), expensive, or that comes with practical restrictions on the use of raw data. Third, data capture systems are easy to write but harder to secure. There are considerable information security problems that would prohibit many simple applications from being used within a secure environment, as in a clinical context. For example, applications are likely to fall foul of UK National Health Service (NHS) information governance principles if they allow one patient to see another's data; transmit patient-identifiable data (PID) over an insecure e-mail network or *via* an unencrypted network link; use inappropriate cryptographic algorithms; fail to prevent unencrypted PID being backed up automatically from a tablet to commercial “cloud” storage; or use servers hosted on insecure or third-party computers, including those in prohibited jurisdictions (13–18).

We describe a novel client–server software package, the Cambridge Cognitive and Psychiatric Assessment Kit (CamCOPS), which attempts to address these problems. It incorporates a number of common and freely available tasks, and can serve as a basis for the addition of further arbitrary tasks in the future. It is an open-source cross-platform system that uses touchscreen tablet devices or desktop/laptop computers for data capture. Instances of the client application (“app”) send their information securely to a central server, owned and controlled by the operating institution. The server provides a “front end” for convenient use by clinicians and researchers, with additional “back-end” facilities to support subsequent research analysis and system interoperability. CamCOPS offers many well-known questionnaires and some more advanced (e.g., animated) tests relevant to cognitive and psychiatric assessment, plus structured and unstructured clinical record-keeping facilities. Data capture can be performed with the app offline, so the system can be used in places with no network reception, such as on domiciliary visits or in unusual radiofrequency environments. The system is compatible with UK NHS information security standards, though compliance with those standards requires other institutional practices as well. As the system is free and open source, we suggest it is suitable for others to use and extend.

## Design and Facilities From a User's Perspective

### Client–Server Architecture

**Figure 1:**
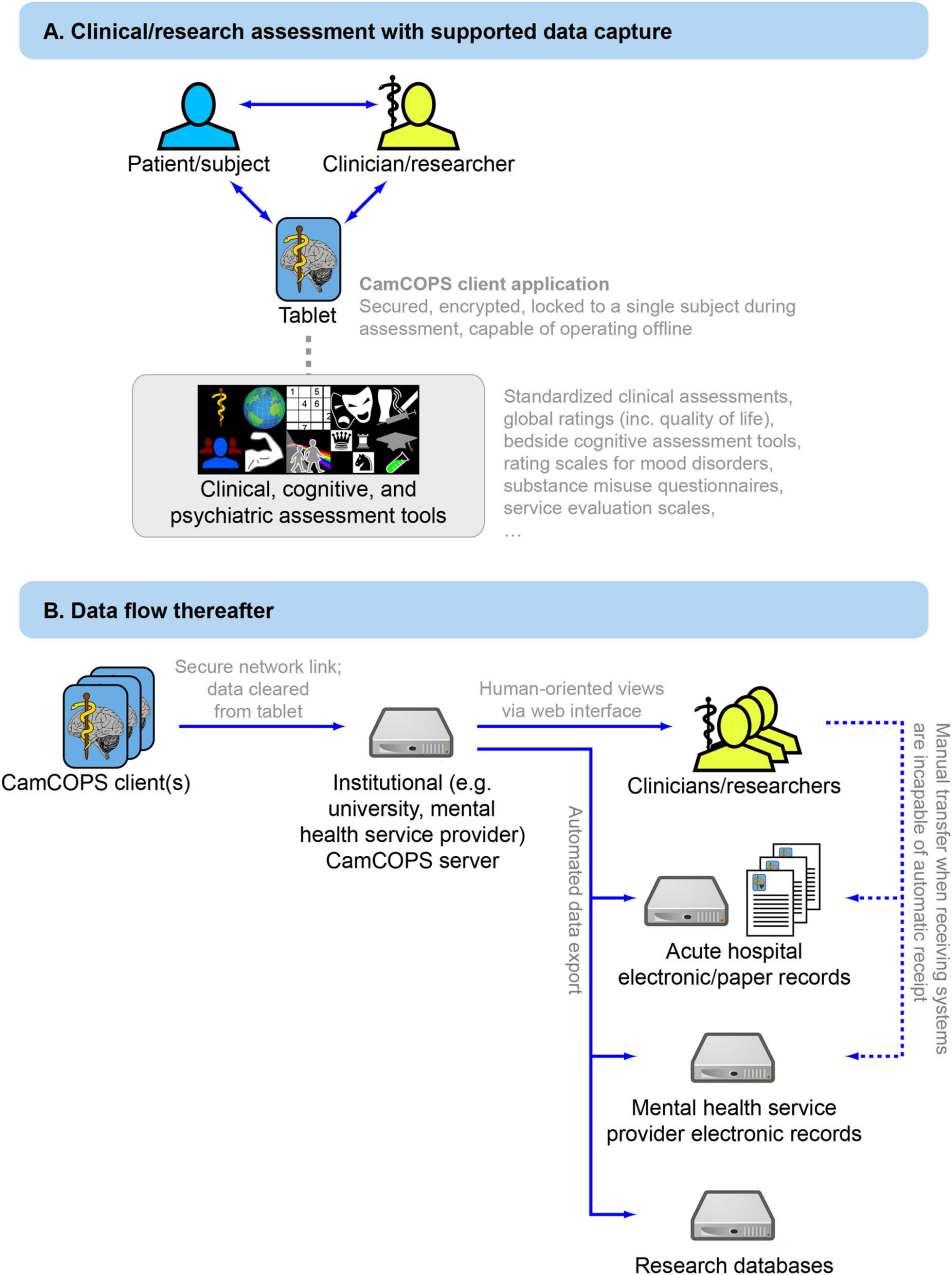
Overview of the CamCOPS data capture system. **(A)** Data capture to the mobile app. **(B)** Subsequent data flow from the mobile device to the institution's CamCOPS server, and thence to individuals viewing or analysing the data, and/or electronic and (if required) paper clinical records.

Data collection and storage is organized around a client–server model (Figure 1). Tablet devices or desktop/laptop computers running the CamCOPS app act as one type of client. A clinician/researcher, a patient/subject, or both together can interact with the device to capture information. Upon request, the app then sends these data securely to the server, located within the host institution. The other main type of client is a clinician/researcher using a web browser or other interface to retrieve information from the server. Strict controls, described below, govern the exchange of data between clients and the server.

### Subject Identification

The software system is designed to cope with clinical environments that use fully identifiable patient information, and with research environments in which participants are assigned a pseudonym or code and an individual's identity is not obtainable without additional information (the pseudonym-to-identity mapping) stored securely elsewhere. The flexibility to operate in both these environments is achieved by defining the meaning of multiple identification (ID) numbers and specifying the minimum and/or maximum information permitted.

The system defines the following subject identity fields, not all of which need to be used: forename, surname, date of birth, sex/gender (M/F/X) (19), and an arbitrary number of ID number types (e.g., national ID number, hospital ID number, study ID number), plus optional address, e-mail, general practitioner, and “other” details for convenience. The administrator defines the meaning of each of the ID number types. CamCOPS supports data verification for some specific ID number types, such as NHS numbers, which incorporate a checksum.

CamCOPS supports two types of ID policy: an *upload* policy and a *finalizing* policy. The upload policy defines the identity information required for the client app to send data to the server. The finalizing policy defines the identity information required for the app to move data to the server, allowing erasure from the client device (with permanent storage on the server). This two-stage process allows data to be entered for new subjects before that subject is registered on a host institution's systems. Two examples may serve to clarify.

In a research environment using pseudonyms, the administrator might define the meaning of “ID number type 1” to be “Research ID.” The upload policy might be “sex AND idnum1”. The finalizing policy might be identical. Therefore, the system would accept uploads only if the researcher had entered a subject's sex/gender and research ID number (as defined by the institution or individual research study concerned), but would not require any other information. Indeed, other information might be prohibited, such as “sex AND idnum1 AND NOT (forename OR surname OR dob)”.

In a complex clinical environment using fully identifiable records, such as a mental health Trust that operates its own patient numbering scheme but also provides urgent on-call services to several hospitals in its region, the administrator might define “idnum1” to mean “Hospital A number,” “idnum2” to mean “Hospital B number,” “idnum3” to mean “NHS number,” and so on. Suppose Hospital A is the provider institution. The upload policy might be “forename AND surname AND dob AND sex AND (idnum1 OR idnum2 OR idnum3)”, and the finalizing policy might be “forename AND surname AND dob AND sex AND idnum1”. This would mean a clinician could enter patient details in Hospital B, using Hospital B's number, without yet knowing the number used by their core institution (Hospital A). The system would require a full name, date of birth, sex/gender, and at least one ID number. At that early stage, the clinician could upload the data, and store a properly identified electronic copy in Hospital B's electronic or paper records. On return to their base in Hospital A, the clinician could look up the patient's number in Hospital A's system or register a new patient, and complete the record by filling in the Hospital A number (idnum1). At this point the software would allow the record to be re-uploaded and deleted from the tablet.

### Using the CamCOPS Client Application to Capture Data

**Figure 2:**
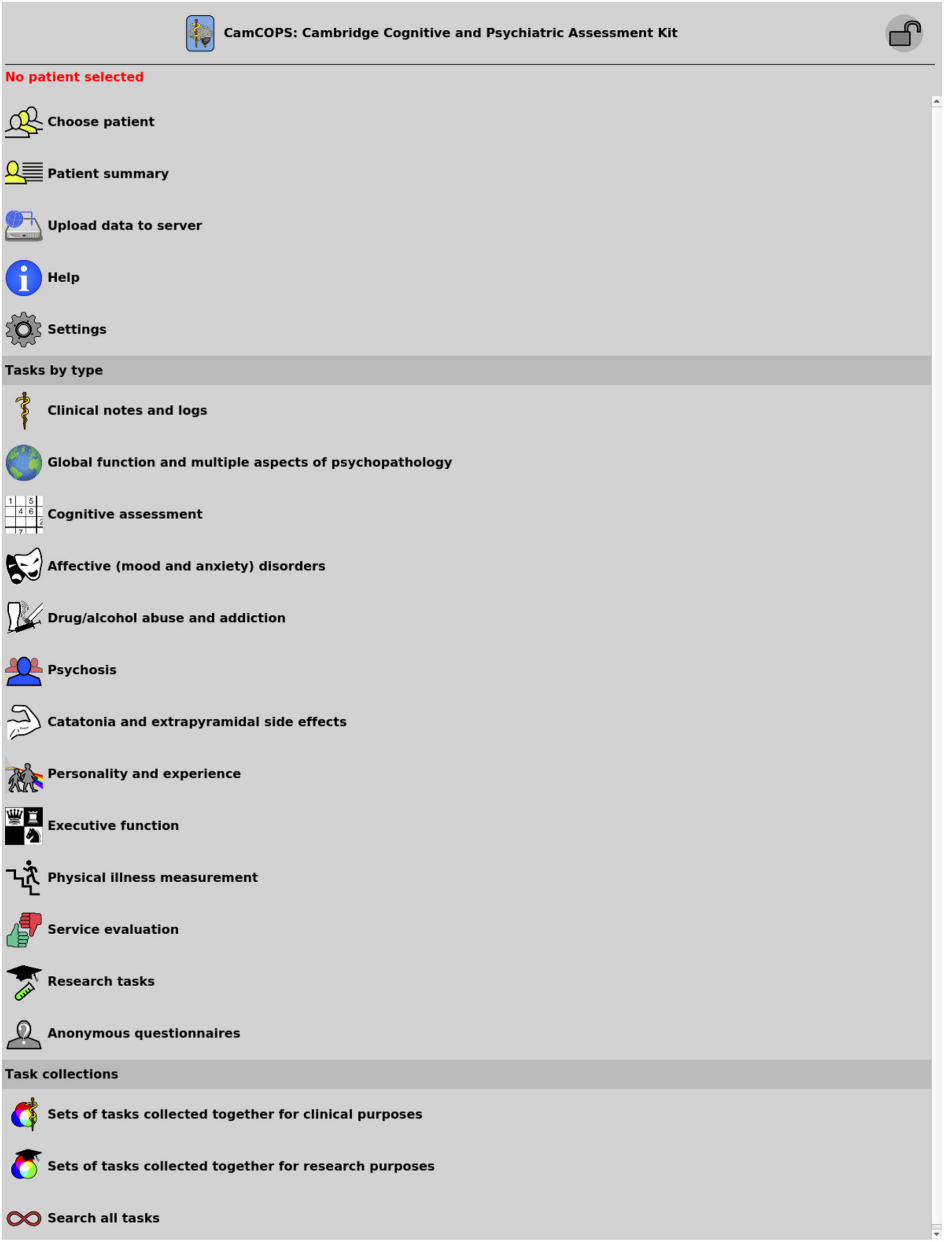
The main menu of the CamCOPS app.

**Figure 3:**
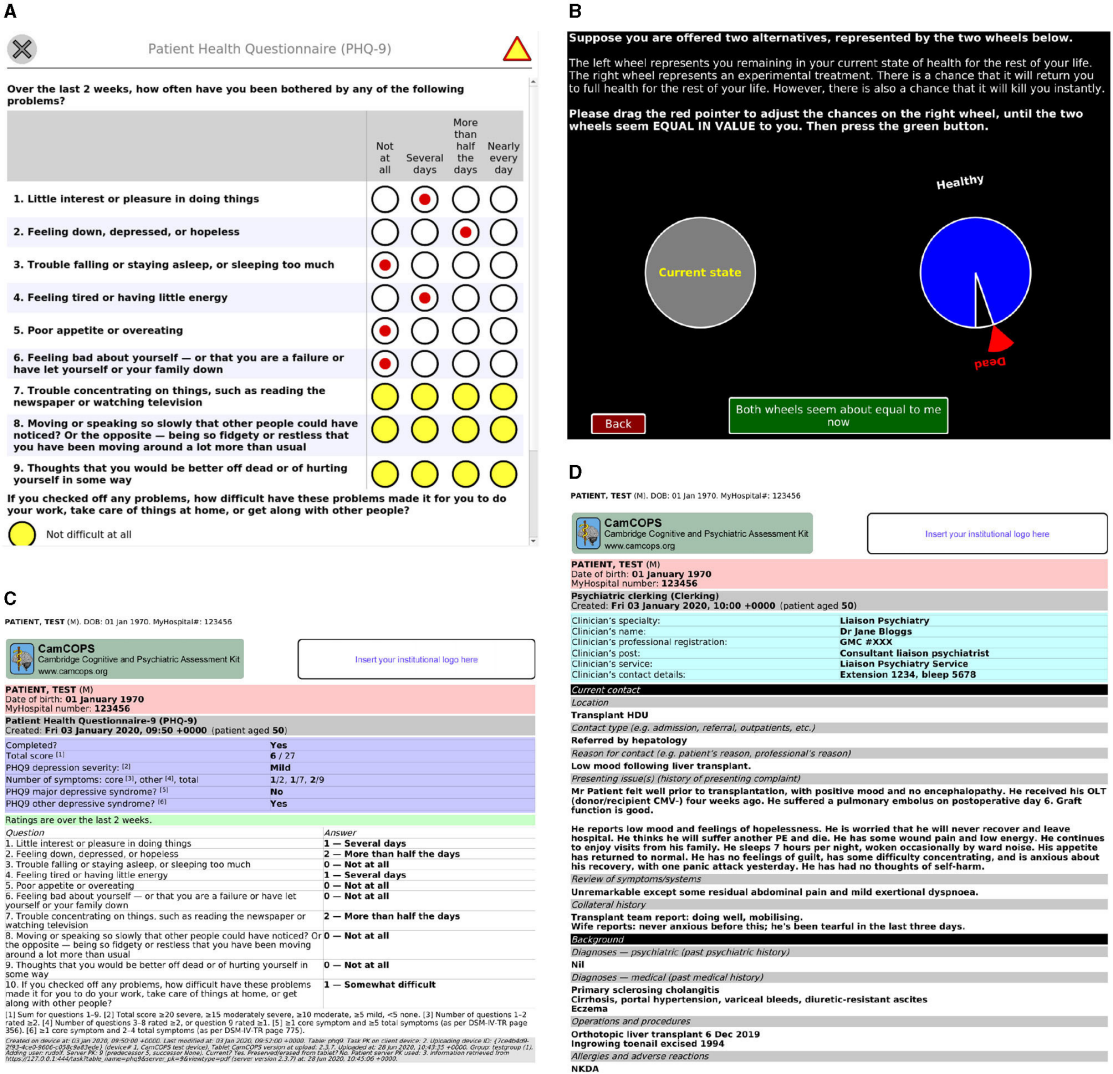
Task examples. **(A)** The PHQ-9 task, a simple questionnaire. Yellow is used to highlight mandatory fields that are not yet complete. **(B)** A simple graphical task: a quality-of-life standard gamble. **(C)** The PDF automatically generated by the server for the task in **(A)** after it was subsequently completed. **(D)** A server view of a task capturing text from an initial psychiatric assessment. All data are entirely fictional. CMV, cytomegalovirus; GMC, UK General Medical Council; HDU, high dependency unit; NKDA, no known drug allergies; OLT, orthotopic liver transplant; PE, pulmonary embolism.

The starting point of the client app is shown in Figure 2. To capture data, the clinician/researcher usually begins by selecting a subject, recording the subject's details according to the identification policies in place. The operator then selects a task and creates a new task instance [current available tasks are listed at (20)]. The task will then run. Typical tasks appear as single-page or multiple-page questionnaires, or animated tasks (Figure 3). They range from very simple tasks, such as the Patient Health Questionnaire-9 [PHQ-9; (2)], through tasks with more complex logic, such as the Clinical Interview Schedule—Revised [CISR; (21, 22)], and those with a more complex interface, such as the Addenbrooke's Cognitive Examination—III [ACE-III; (3)], to complex animated tasks such as a three-dimensional intradimensional/extradimensional set-shifting task (23).

Questionnaire-style tasks have a consistent user interface, indicating mandatory/optional data items and permitting progression when mandatory information has been provided. The questionnaire user interface is consistent across platforms (operating systems, OSs). User customization of the interface is limited but includes font sizing for accessibility and language selection (discussed further below).

Some tasks are intrinsically anonymous, in which case they are not associated with any subject information at any stage, visibly or invisibly.

Tasks may collect information from the patient/subject alone, the clinician/researcher alone, or both together. Questionnaire-style tasks provide consistent colour-based visual cues as to the respondent. Tasks that involve the clinician's/researcher's judgement also record the details of the clinician/researcher conducting the assessment. These details may be pre-configured by the operator in advance so they are automatically entered, but may be edited, for example when a tablet-wielding clinician needs to document an assessment conducted by a more senior clinician. Some OSs (e.g., iOS, Android) are not designed for multi-user use, and the CamCOPS client does not offer specific multi-user facilities, but it stores per-user data when running under multi-user OSs.

CamCOPS also supports multimedia facilities in direct and indirect ways. Sound is used in some tasks, and the app can use the device's camera to capture photographs (such as of handwriting or other paper notes). In addition, text fields in CamCOPS can accept input from voice-recognition dictation systems supported by the OS.

The operating mode described above is oriented towards interactive use by a clinician/researcher and patient/subject together or consecutively (“clinician mode”). In addition, CamCOPS also supports a “single user” mode. This is intended for patients/subjects to complete tasks by themselves, for example at home in advance of a clinic appointment or between appointments, or as part of an ongoing research study. To use this mode, the clinical or research team defines one or more task schedules on the server (such as a weekly PHQ-9 for 6 weeks), and registers the subjects. The subjects download the CamCOPS app and enter the server's URL (uniform resource locator) with an identification/security code. The app is presented *via* a highly simplified user interface, and will then offer tasks to the subject automatically according to the defined schedule(s), moving data to the server whenever a task is completed.

### Viewing Completed Tasks

Once complete, tasks may be viewed on the client device (e.g., tablet) or the server. Tasks are visible on the tablet until they are moved off it (typically at the point of upload to the server) and are visible to authorized users on the server as soon as they have been uploaded, and indefinitely thereafter.

In the client app, tasks display summary details, such as the total score from a questionnaire, and often also a read-only facsimile of the full task, as seen by the subject or clinician during the task. The facsimile view is provided automatically for all questionnaire-style tasks.

On the server, tasks provide an HTML (hypertext markup language) view, optimized for browsing speed, or a PDF (Portable Document Format) view, optimized for printing (Figure 3). Both show the raw captured data, plus summary information calculated automatically. The PDF view adds subject identification information to all pages, making them suitable for printing and direct use in paper-based clinical environments, and provides space for an authenticating physical signature where tasks have been conducted by clinicians (as opposed to tasks that are entirely self-rated by subjects).

The user may filter tasks by subject, date, task type, and so on, but may also search also by free-text content; thus, for example, all task instances containing the word “overdose” can be searched for, whether those “tasks” relate to initial psychiatric assessment, a progress note, or a comment made by a participant in a research questionnaire.

**Figure 4:**
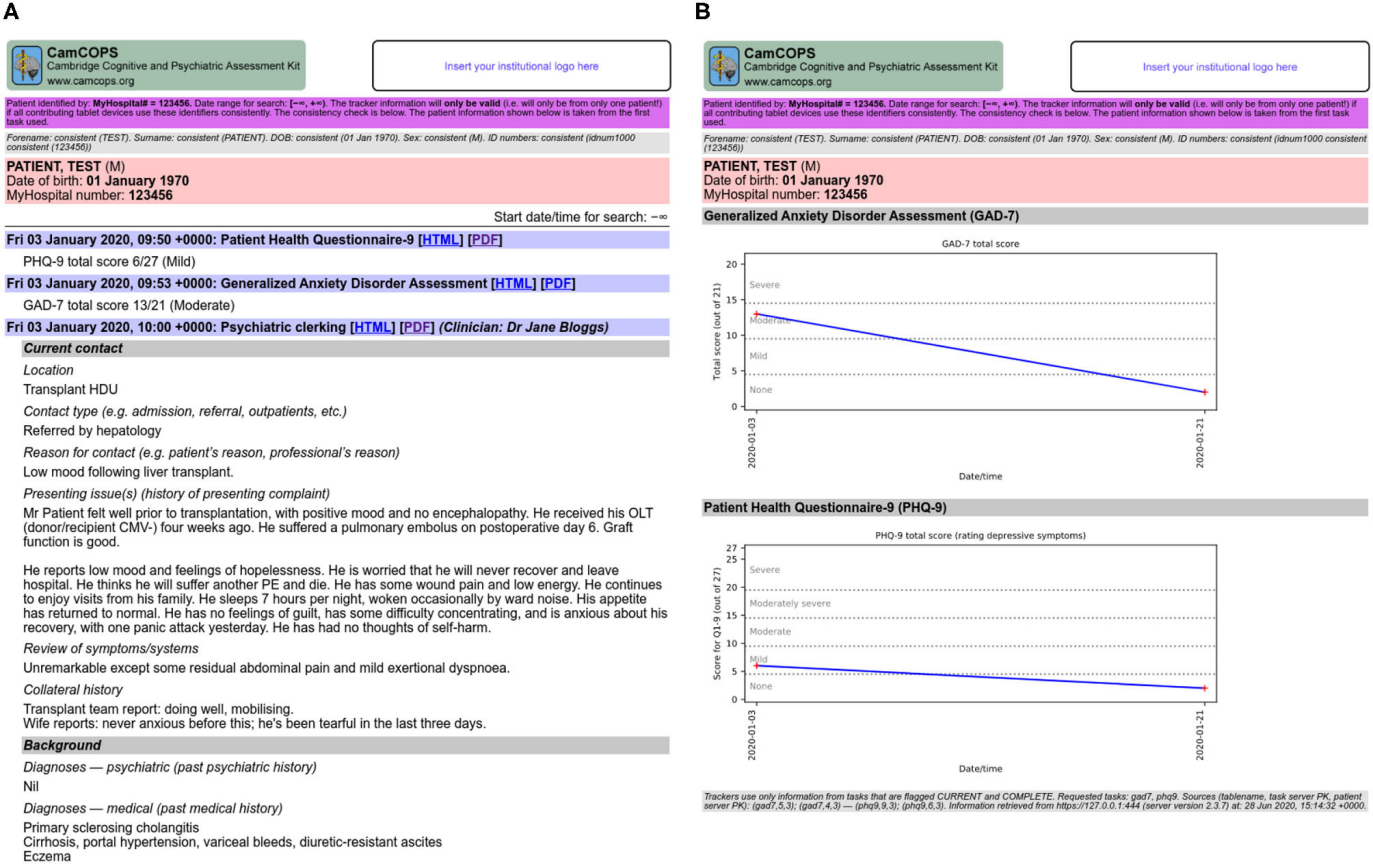
Some multi-task views provided by the server. **(A)** Clinical text view. **(B)** Numerical tracker view. All data are fictional. CMV, cytomegalovirus; HDU, high dependency unit; OLT, orthotopic liver transplant; PE, pulmonary embolism.

The server also provides a summary view oriented towards text, and another oriented towards numerical data (Figure 4), both available in HTML and PDF format. The clinical text view shows all tasks for a given subject, optionally constrained by date, and shows key text from each task (e.g., summary scores for cognitive assessments or mood questionnaires, or all text for clinical assessments and progress notes), with hyperlinks to the full tasks for further detail. The numerical trackers show trends in numerical information over time in graphical format (such as for mood disorder questionnaire summary scores, or body mass index [BMI]).

### Task Information

The online documentation (20) includes help pages for all CamCOPS tasks, hyperlinked to from the app itself. The help pages include details of each task's history and provenance, with links to key studies such as on the task's validity and reliability, where applicable. It remains for end-user clinicians/researchers to establish the applicability of a particular task to a given subject/patient in their context.

### Internationalization

Text used by the client and server software is internationalized, supporting arbitrary languages (with current text for English and Danish), and the task framework supports internationalization of individual tasks. Where tasks supported by CamCOPS have been translated and that translation validated, the framework permits the translated versions to be selected automatically. Users choose their preferred language dynamically.

### Interfaces for Research

While clinicians typically focus on a single patient at a time, researchers typically analyse data from multiple subjects together. The CamCOPS server allows suitably authorized users to download data in bulk, for exporting to other databases or manipulation in spreadsheets or statistical software. Download formats include Microsoft Excel (XLSX), OpenOffice/LibreOffice (ODS open document spreadsheet format), tab-separated values (TSV), R script (24) (though R can also read a number of other structured formats exported by CamCOPS), textual SQL (structured query language), and binary SQLite format (25). In addition, users can view raw and calculated data in structured XML (extensible markup language) format. Administrators may export data in bulk, including *via* formats suitable for third-party anonymisation tools (26).

Following the DRY (“don't repeat yourself”) principle of software engineering (27), CamCOPS stores raw data, not calculated data. For example, PHQ-9 information is stored as a set of answers to each of the 10 questions; the summary scores are not stored but are calculated “live” upon request. BMI information is stored as height and mass, and so on. The method of calculation of summary scores is specific to each task. To simplify research and to reduce errors caused by researchers having to calculate summary scores, CamCOPS calculates these. The system offers a basic research data dump oriented towards convenience, in which most tasks provide a single spreadsheet-style page. This has one row per task instance, including includes raw data, summary scores (calculated at the moment of request), and subject identifiers together.

**Figure 5:**
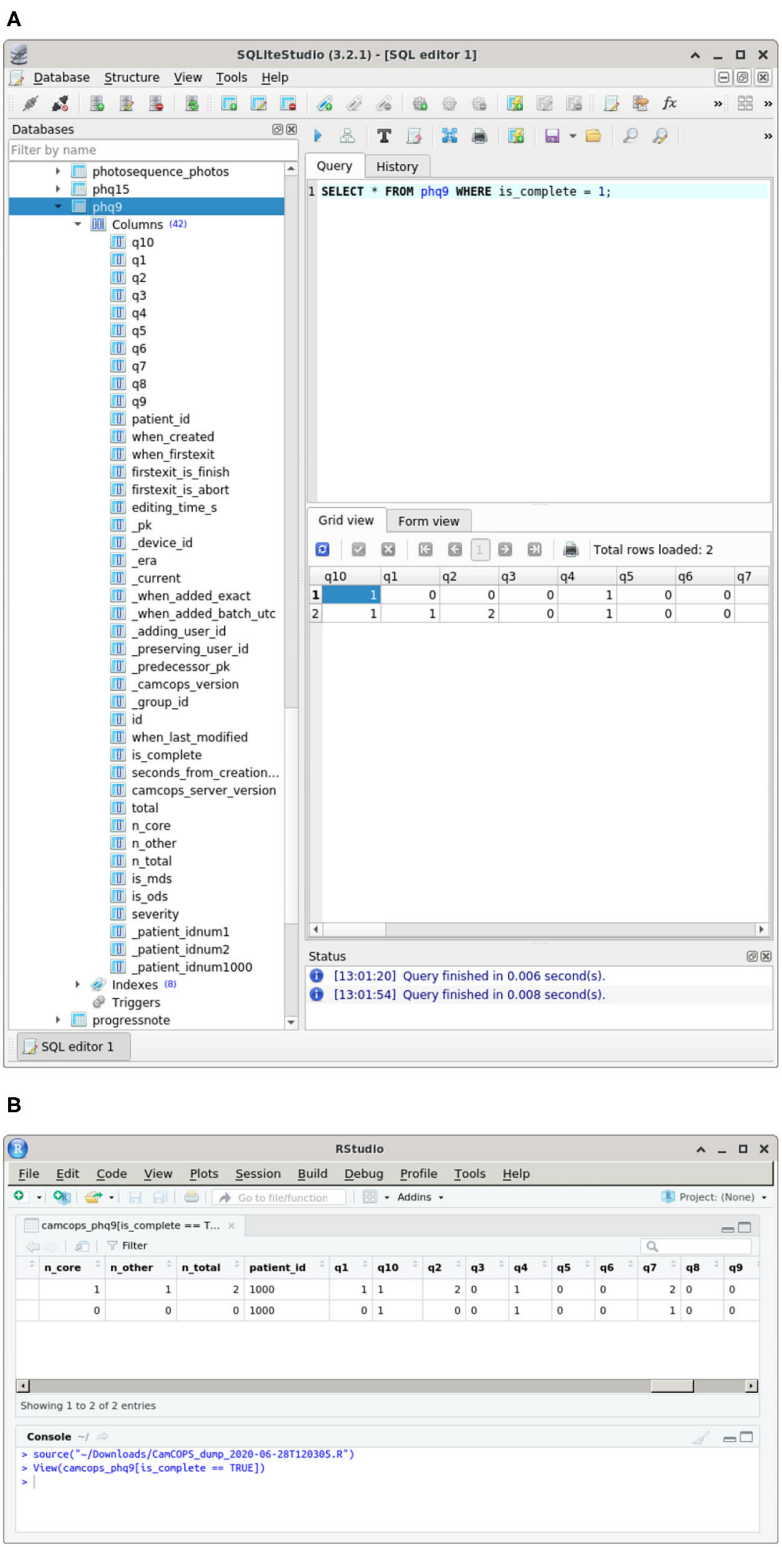
Some research-oriented methods of data access. **(A)** Structured data in a relational database downloaded from CamCOPS. An SQLite database (25) is shown in SQLiteStudio (28). **(B)** Data downloaded and imported directly into R (24), shown inside RStudio (29). All data are fictional.

The CamCOPS server is an interface to a relational database with a well-defined structure. It is conceivable—but in our view highly unlikely—that institutions would wish to give users direct access to this database (which would circumvent standard security controls). However, for full access to relevant raw data, CamCOPS allows authorized users to download a relational database containing data of interest to and permitted to them, as well as downloading structured data directly to statistical packages such as R (24) (Figure 5). Relational database export is more powerful but more complex for users. Summary scores are also provided automatically in this situation, by calculating them as the download is created.

### Group System

A given institution may need to capture data in several different contexts. For example, it might provide a number of clinical services. Staff in those services might want to analyse their service's data in isolation but also see data for their patients that has been collected by other clinical services. Simultaneously, the institution might support clinical research using identifiable data. Researchers might typically be allowed to see only the data collected for their subjects as part of their study (while, simultaneously, clinicians looking after those patients might want to see any clinically relevant data, collected as part of the research or otherwise). Finally, the institution might support research using pseudonymised data.

CamCOPS supports these usage scenarios simultaneously via groups. A group might represent, for example, a clinical service or a specific research study. Users belong to one or more groups, and upload data into a specific group at any one time. A group has its own set of ID policies (as above), and may, as a whole, be permitted to see data from specific other groups. Thus, for example, a clinical group might use fully identifiable data according to a certain identification standard, while a research group may use a study-specific pseudonym and prohibit direct identifiers. A researcher might belong to one or more research study groups, and only be permitted to see data collected within them. A different clinical research group might use an ID number type in common with clinical services, and the system can be configured to allow clinicians to see data from all clinical services plus “research” data for the same patients, without researchers being able to see “clinical” data.

While is also possible to run multiple instances of the CamCOPS server, the group system is intended to make this unnecessary for most purposes.

### Export Facilities

Individual users may wish to download different subsets of data in various formats (as above), but it may also be desirable to export data systematically from the server. A prototypical example would be the need to copy clinically relevant data to an institution's primary EHR system. CamCOPS supports export in different formats (including PDF, HTML, and XML) and *via* different transmission methods [including *via* HL7 (30), e-mail, and file-based export]. Exports can be scheduled and/or triggered by the arrival of a task on the server. CamCOPS also supports direct export to relational databases, and to REDCap (31) via an open-source interface (32). We are also seeking to improve integration with other EHR systems, via standard information exchange methods such as FHIR (33).

### Other Administrative Operations

Subject to permission, users can run reports on the server. These include activity reports and search tools. Group administrators can manage users within their groups, and superusers have full control over the whole system.

To assist compliance with NHS records management procedures (34–37), specific records can be erased of content or deleted entirely by privileged users. All records for a given patient can be deleted entirely, as might be required after a certain number of years have elapsed, or in a research context if a subject withdraws consent. Records can be annotated manually by users with annotation authority (for example, to indicate an error or that the patient disputes its contents) and patient details can be corrected (for example, if a name was misspelled).

## Implementation

### Software Platforms

The client app is written in C++ (38) using the open-source Qt cross-platform framework (39). CamCOPS has been used on Android devices, iOS devices (e.g., Apple iPad), Windows tablets, and conventional desktop computers (Windows, Linux, macOS). Application data is stored in an encrypted database using SQLCipher (40), based on SQLite (25). Cryptography is provided by OpenSSL (41), developed from SSLeay (42).

The CamCOPS server is cross-platform software written in Python (43). It is supplied with HTTP (hypertext transfer protocol) servers including CherryPy (44) and Gunicorn (45), which may be used directly or *via* a more sophisticated web server such as the Apache HTTP Server (46). It is normally run under Linux (47) (tested with Ubuntu/Debian and CentOS). CamCOPS typically uses the open-source MySQL/MariaDB database (48, 49) but supports others *via* SQLAlchemy (50). A Docker Compose containerized application is provided for consistency and ease of installation (51).

### Distribution

Documentation is online (20). The source code and some binaries are available from GitHub (52). The Android client app is available *via* the Android Google Play Store, and the iOS version *via* the Apple App Store. Apple prohibits public distribution, by other routes, of applications that can be installed on arbitrary iOS devices (53).

### Data Storage and Synchronization

CamCOPS stores its data using standard relational database mechanisms (54). A simple format is used, with a table to record subject details, a linked table to record ID numbers, and one or more tables for each task, linked to the subject table except in the case of anonymous tasks. The app records the time of last modification for all records. Tasks also record their creation time, the time the task was first exited, and whether the task was completed or aborted at that time. This allows measurement of the time it takes to complete a task. Dates and times captured by tasks are stored in ISO-8601 format, with time zone information and arbitrary temporal precision (by default accurate to 1 ms to allow reaction time recording). Binary large objects (BLOBs) such as images are stored in the database; this is not definitively better or worse than storage in a filesystem (with the database holding a reference to the file), but storage in the database has the advantage of being easily ACID (atomicity, consistency, isolation, durability) compliant.

Subject identification is one area where CamCOPS departs from the DRY principle (27). The CamCOPS server maintains copies of each device's patient identification records, as of the moment of each upload. It does so because repetition is a key safety feature to ensure correct patient identification in clinical environments, and because the use of additional non-unique identifiers is important for clinical safety. For example, if clinical records only had NHS numbers on them, they would be technically correct but clinically useless, because clinicians think of patients by their name. CamCOPS does not fetch other details; for example, when given an ID number, it does not fetch a patient's forename/surname from a national or institutional database. Therefore, users need to enter this information. Of course, several CamCOPS users can enter data about the same patient on different devices, and it is possible for users to enter incorrect name/number combinations or to misspell names. The appropriate logical mechanism to link multiple records about the same patient is defined by the host institution, but is typically by the use of a single standardized institutional or national ID number. When the CamCOPS server interface combines records, linking them by the desired method (e.g., institutional ID number), it warns the user prominently if any records contain incompatible information (e.g., misspelled names or non-matching dates of birth). Suitably authorized users can correct mistakes (e.g., misspelled names) on the server, once records have been finalized to the server. CamCOPS contains framework code to support validation of subject identity at the point of upload (e.g., against an institutional database), but this has not been used concretely yet.

No history information is stored in the client app's database, but history information is added by the server. Servers distinguish records from different client devices using a unique device identifier. The server also marks uploaded records with a Boolean “current” flag. When a record is re-uploaded, the old record is marked as no longer current, linked to its successor, and its time of removal and removing user recorded, while the new record is marked current, linked to its predecessor, and its time of addition and adding user recorded. This allows a modification history to be followed, and permits linking of contemporaneous information across multiple tables.

The client app can copy data to the server, but may also move data by uploading it, wiping it from local storage, and starting afresh. A “move” may be accomplished for individual anonymous tasks, for all tasks associated with a particular subject or subjects, or for all data on the device. Optionally, basic subject identifiers can be preserved on the device to speed the entry of subsequent data for the same subject. The server manages this “move or copy” capability by adding a further “era” field, which is either the string literal “NOW” (for records still present on the device) or the date/time that the data was uploaded and wiped from the device. Using these mechanisms, which allow the server to store multiple snapshots of a device's state over time, records can be wiped from the device yet remain available on the server, or be modified and “overwritten” on the server, leaving a historical trail of modifications available for inspection.

Uploads are accomplished as atomic transactions; that is, they succeed in their entirety or fail as a whole. This preserves the relational structure of the database in the face of unexpected network disruption.

### Hardware Platforms and Costs

CamCOPS has been used on tablets, touchscreen laptops, and conventional laptop/desktop computers. In practice, we have found that hardware keyboards (e.g., Bluetooth keyboards for tablets) are essential for any form of data capture that uses text extensively, such as clinical note-taking, because on-screen keyboards are slow to operate. The choice of tablet may depend on price, on the form of network connectivity desired (e.g., Wi-Fi only vs. Wi-Fi plus 3G/4G cellular data), and on the software distribution model desired. For example, Android tablets can install software from the Google Play Store, but can also install software downloaded from arbitrary web sites. Thus, an institution could download the CamCOPS code, modify it for its own purposes, compile it using the open-source development tools, and distribute it on its own internal or public-facing web site. In contrast, distribution to iPad devices is only permissible *via* the Apple App Store or via internal distribution by organizations or individuals who pay for the Apple iOS Developer Program (53, 55).

CamCOPS is free of charge, but the system as a whole requires some infrastructure. In a university research environment, a simple server installation requires only a single Linux physical server or virtual machine with a network connection, plus a transport layer security (TLS)/secure sockets layer (SSL) X.509 certificate (“SSL certificate”) for secure HTTP (HTTPS). In a UK NHS clinical research environment, such a server may need to operate within a secure network, and there may be additional costs for virtual private network (VPN) access to that network from outside. The main additional cost is for client devices, which vary according to user preference (e.g., Android tablet; iPad; Windows tablet; touchscreen laptop). The client devices must be able to communicate with the server (e.g., *via* a wired connection, Wi-Fi, or 3G/4G cellular data).

### Performance

The server is optimized for performance using multithreading or multiprocessing and caching systems. The basic overhead of the scripts is very low: a server with an Intel dual-core 3 GHz processor and solid-state disks took 3 ± 1 ms (mean ± standard deviation) to process an HTTP transaction, retrieve and validate session information from the database, and return the main menu (*n* = 100). Retrieving a PHQ-9 task in HTML format took 9.5 ± 1.9 ms (*n* = 100), including the time taken to audit the request. Registration of a mobile device took 10.6 ± 0.4 ms (*n* = 100) including approximately 6 ms for password cryptography, which is deliberately slow in the *bcrypt* system (56). Performance in practice depends also on the underlying database and hardware; MySQL offers the option to trade full ACID compliance for performance *via* the *innodb_flush_log_at_trx_commit* option (48), set for speed during the benchmarks given above. The client is similarly optimized for performance, including the use of a multithreaded database handler so that encryption does not slow the user interface.

### Writing New Tasks

CamCOPS has >120 tasks and more are regularly added. Many psychiatric assessment scales use a questionnaire style, with multiple-choice fields, yes/no fields, free text, and other common input elements. Other tasks may require significant programming, such as cognitive assessment tasks that present stimuli and measure responses in a time-sensitive or complex way. The CamCOPS platform supports arbitrary tasks by providing a questionnaire-style interface, a *tabula rasa* allowing graphical and arbitrarily complex tasks, or a combination of the two.

Free-form tasks use C++/Qt code to create tasks of arbitrary complexity including visual animations and auditory stimuli. Questionnaire-style tasks use a simpler standardized interface. Questionnaires are built from combinations of elements, including:

- static text, images, lines, and spacing;
- an audio player;
- Boolean fields (NULL/false/true) with associated text or an image;
- a button, capable of executing arbitrary code;
- a canvas for sketching, which can display a background image;
- a countdown, to assist clinicians in timed tasks;
- date, time, and date/time pickers;
- a diagnostic code element, usable with any hierarchical diagnostic code system such as ICD-9-CM (compatible with DSM-IV-TR) or ICD-10 (57, 58);
- multiple-choice (1-from-*n*) questions (MCQs), in a variety of common layouts;
- multiple-response (*k*-from-*n*) questions;
- photographs, taken using the mobile device's camera, also useful for photocopying paper records;
- inline and pop-up pickers (an alternative 1-from-*n* representation);
- discrete and continuous scales represented by sliders;
- a thermometer-style scale;
- fields accepting typed input, with validation for textual or numerical fields;
- containers for laying out other elements.

The software is designed to be extensible. Adding a new questionnaire-style task presently requires (1) a C++ header/source file for the client app, specifying the task's structures and content (see excerpt in Box 1); (2) addition of that task to the app's master task list and menu system; (3) addition of strings to a string file in any languages required; and (4) a Python file for the server, specifying the table structure and the HTML content that is automatically used to make the server's HTML and PDF views.

Box 1 C++ code snippet illustrating the core of the implementation of a questionnaire-style task, the PHQ-9 (2), within the CamCOPS client app. This task uses some static text, a grid-style set of multiple-choice questions (MCQs) for questions 1–9 that all share a set of answers mapped to the data values 0–3, and a single MCQ for question 10. Calls to the xstring() function yield internationalized (language-/locale-specific) task strings; for example, xstring(“q1”) in the English locale evaluates to “1. Little interest or pleasure in doing things,” while xstring(“a3”) evaluates to “Nearly every day.” See Figure 3A for the resulting task.

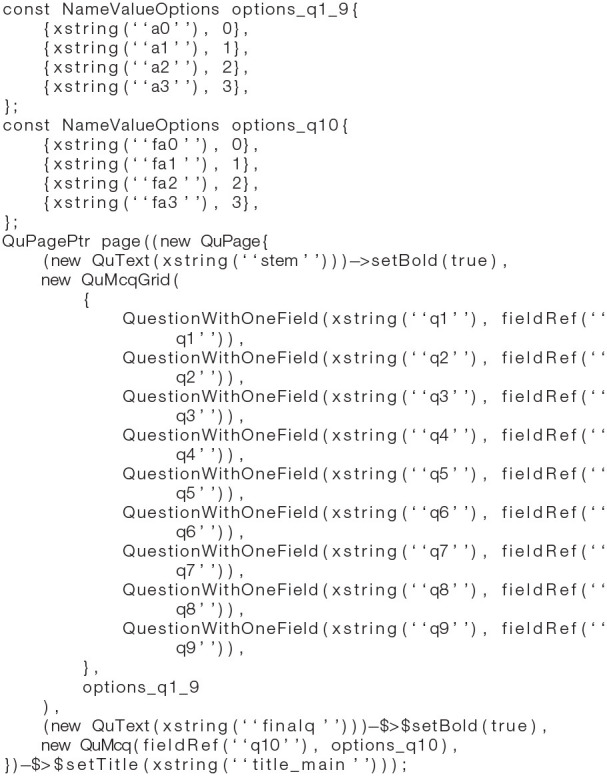

## Intellectual Property Management

The intellectual property in the CamCOPS software must be distinguished from the intellectual property in tasks supported by the CamCOPS platform. The source code for CamCOPS is licensed under the open-source GNU General Public License v3+ (59). The same licence applies to tasks developed *de novo* by us as part of the CamCOPS project. CamCOPS also uses some third-party software libraries (e.g., for cryptography) with open-source licences. We took care to ensure that all other material potentially subject to others' copyright, such as text from tasks developed by others, is not included in the main CamCOPS source code. For example, the code developed by us to present and score a questionnaire is segregated from the text that makes up an individual questionnaire. Furthermore, we have taken care to ensure that all use of tasks within CamCOPS is permitted either by the copyright declarations published with the original versions of the tasks, or by explicit verification for each task. We have not included content for any task where we are aware of copyright restrictions incompatible with distribution under an open-source licence. CamCOPS supports tasks under the following copyright models, ordered from least to most restrictive.

1. CamCOPS includes a number of freely available tasks. For example, the Patient Health Questionnaire-9 (2) is published with an explicit declaration that it is in the public domain (60), while the copyright to the National Adult Reading Test (61) is held by its author, who kindly gave permission for its free use in perpetuity (62).
2. Some tasks are published with a copyright declaration allowing, for example, free non-commercial use and reproduction with appropriate attribution, but restricting commercial use [e.g., (63)]. CamCOPS includes user-completed fields indicating whether the software is being used for clinical, research, educational, and/or commercial use; each field can take the value “yes,” “no,” or “unknown.” It restricts some tasks on this basis, according to their published permissions. These tasks cannot then be used outside their copyright restrictions without explicit dishonesty by the user, in breach of the CamCOPS terms and conditions of use that all users must acknowledge, and of the tasks' licensing terms. However, it remains the user's responsibility to check that they are legally permitted to use each task, and to comply with any licensing terms.
3. Some tasks allow reproduction for institutions that have paid a license fee or undergone another registration process, but not otherwise. To cope with these, CamCOPS supports a method where the default task is only a data collection tool (as for type 4 below), with copyright-free placeholder strings such as “Question 1.” The institution may then choose to install an XML file containing the actual task text on their server instance(s). When the CamCOPS client app registers with the server, it downloads any strings specific to that institution. As these add-on XML files are not distributed with the CamCOPS itself (merely templates), the open-source licensing of CamCOPS does not conflict with the restricted licensing applicable to such tasks. Responsibility for any add-on files rests with the hosting institution, as does compliance with any licensing terms, including any training requirements.
4. In addition, we had a local need to capture information electronically for tasks that are distributed commercially and cannot be distributed under an open-source licence, such as the Beck Depression Inventory (64). For this situation, in an attempt to improve on the research method of typing data by hand into a spreadsheet, we developed “skeleton” questionnaires that refer to the original questions only as “Question 1,” “Question 2,” and so on. This method allows data to be recorded electronically without including elements subject to copyright, but makes the task implementation useless except to clinicians/researchers who can refer to their own licensed copy of the test.

We note that ascertaining copyright status can be difficult, particularly for older tasks. For example, the Edinburgh Postnatal Depression Scale was published with a notice saying “users may reproduce the scale without further permission providing they respect copyright by quoting the names of the authors, the title and the source of the paper in all reproduced copies” (65), but this instruction has been superseded by a different set of permissions that prohibit unrestricted electronic reproduction (66). In all instances, if we have inadvertently erred in our assessment of a task's copyright status or licensing permissions, we will remove it from CamCOPS with our apologies if we are alerted to the fact.

## Information Governance, Security, and Audit

The CamCOPS information governance and security model is multi-layered. It is not sufficient to have a “secure” mobile application; a hosting institution must implement other security measures.

### Minimizing Patient-Identifiable Data Held on Mobile Devices

Assuming that identifiable information is used at all, there are two main methods by which the CamCOPS app minimizes the amount of patient-identifiable information held on a mobile device.

First, data exchange with the server is essentially one-way (upload, not download). Therefore, even if all the security measures (see below) were somehow circumvented, possession of a device implies possession of information about at most a few patients, created recently on that device. The app will not retrieve information created on other devices.

Second, its dominant method of uploading is to *move* data to the server, not to *copy* it. Users upload when they choose, and can be prompted whenever a new task is complete. When they upload, they are offered a three-way choice. (1) The “move” option moves details of all patients and their task data to the server, deleting that data irreversibly from the device. If some patients do not meet the server's finalizing criteria, as above, then the user cannot move data until this problem is fixed. (2) The “move, keeping patients” option moves all patients' task data, but it keeps the basic patient details, so the user can add more tasks for these patients later. (3) The “copy” option copies data to the server, though it still “moves” patients or anonymous tasks that the user has explicitly marked as “finished.”

Users are encouraged to move data whenever possible. However, the option to copy remains important, as in the multi-hospital example given above: when a patient has been entered using institution B's ID number, information must be uploaded and stored in institution B's records immediately, but institution A's number must later be added before that record can be finalized and moved to the server.

### Device Security

Mobile device security is provided without the need for users to encrypt the entire device, since they might inadvertently fail to do so. All CamCOPS data is stored using the 256-bit form of the Advanced Encryption Standard (AES) cipher suite (AES-256) (67).

As a general security feature, not specifically related to or required by CamCOPS for its security, users may also choose to encrypt their devices using a strong password. Android devices allow on-device encryption (for Android version 3 and higher). This encrypts applications' data areas with a passcode (68, 69). Apple iPads and related iOS devices invoke encryption when a passcode is entered (70, 71). Both these platforms have “sandboxes” to prevent one application seeing another's data (71, 72). After device encryption is enabled, the tablet device will require a passcode every time it is turned on or re-activated after its screensaver has activated. Since a misplaced tablet will lock itself, lost or stolen tablets become useless to anyone except their owner. Other OSs provide similar functions.

### Application Security

The CamCOPS app has three security modes when running in “clinician mode”: Locked, Unlocked, and Privileged. In the Locked mode, the app is locked to a single subject and can only view or add records pertaining to that subject, or anonymous tasks. This mode is designed for a clinician/researcher to hand the device to a subject. It takes a single touch to lock the app, but it takes a password to unlock it. In the Unlocked mode, all data may be viewed and edited. This mode is designed for use by clinicians/researchers. Privileged mode is designed for administrators' use. In Privileged mode, features such as the following are unlocked: configuring the link to a server, registering the device with a server, and (if the device permits) exporting the local database to an insecure storage area such as a removable secure digital (SD) card. (Despite the name, there is nothing intrinsically secure about an SD card.).

CamCOPS requires the app password to start, and to access the encrypted databases. Since data security is prioritized, there is no recovery method if this password is lost: the app would require re-installation, with loss of any data not yet uploaded.

In typical clinical use, an administrator might set up CamCOPS to point to the appropriate institutional server and then give clinicians the “unlock” password but not the privileged-mode password. This would not be impossible for an astute clinician to circumvent, by uninstalling and reinstalling the app, but the clinician is, after all, entrusted with the primary clinical information in any case. In practice, this extra level of security may help to prevent the clinician from misconfiguring the app by accident.

Internally, the app never sends patient-identifiable data to the device's system logging stream, except when authorized *via* a privileged-mode data dump, so a malicious user who plugs the device into a debugging computer, such as via a Universal Serial Bus (USB) cable, will not see patient-identifiable data that way. The CamCOPS app stores its stores its “unlock” and privileged-mode passwords using irreversible *bcrypt* hashes (56)—that is, the passwords themselves are never stored. Moreover, the database in which these hashes are stored is itself encrypted. The administrator may choose, following local institutional policy, whether the CamCOPS app stores the user's server password using reversible encryption or does not store it at all. Storage with encryption is more convenient but less secure, since the password would be potentially vulnerable to a skilled attacker in possession of the CamCOPS app password (and the device's unlock code, if enabled). Not storing the password is more secure, but requires the user to enter the password each time data is uploaded.

### Network Link and Server Security

Communication between the client app and the server is secured as follows. The app's network link to the server is constrained to use HTTPS and therefore link encryption. The specific encryption used depends on the web server's configuration; typically, it would be configured to use TLS 1.2 with the AES cipher suite (73). By default, the app will insist on a validated SSL certificate, though this can be turned off by the administrator for low-security environments that use a self-signed (“snake oil”) SSL certificate.

Client application instances must register with a server. This serves several purposes. Firstly, the server does not want unauthorized devices uploading to it. Therefore, the server will only accept uploads from registered devices, and requires users to authenticate, with a username previously approved by an administrator for device registration, before accepting registration. Secondly, administrators will not want their clinicians or researchers to upload data to unauthorized servers. Registration is therefore a privileged-mode function. We envisage that in practice, device registration would be managed by an administrator for high-security environments. Thirdly, the server and the app should share a set of ID descriptions and upload/finalizing policies (see “Subject identification” above). The app reads the ID descriptions and policies from the server at registration, and re-checks these before commencing an upload.

The server requires username/password identification before it will accept an upload, and requires that the device be validly registered. Devices are distinguished by a unique device identifier (a long random number). The server accepts incoming data but will not provide unrelated data to the app. Therefore, even a hand-crafted app masquerading as an instance of CamCOPS and in possession of a valid username, password, and device ID cannot download sensitive data *via* the app–server link. The server will not add new fields or tables based on the claims of the uploading agent, and will not upload to reserved tables or fields. The server takes standard precautions against SQL injection (74).

Communication between users and the server *via* the web front end is secured as follows. The web front end is constrained to use HTTPS and therefore link encryption. This requires appropriate configuration of the web server hosting the CamCOPS installation, but is also ensured by CamCOPS through its session security methods. Access is governed by username/password pairs. The server stores all CamCOPS passwords using irreversible hashes (56); passwords themselves are not stored. The only session information stored on the client side is a HTTPS-only session cookie containing a server-generated session ID and token; the token is regenerated by the server at login to prevent session fixation (75). Sessions expire after a defined period of inactivity and cannot be transferred between client Internet Protocol (IP) addresses. Administrators configure a maximum password lifetime. The server will lock user accounts for increasing periods of time in response to multiple login failures. It will mimic normal login failure behaviour for non-existent usernames, including the time it would normally take for password cryptography, to prevent automated username discovery. Optionally, administrators may require multi-factor authentication, such as *via* e-mail, text message (short message service, SMS), or a third-party authenticator app (e.g., Google Authenticator).

Internally, the server must deal briefly with a clear-text database password, but encapsulates all such code with an error-trapping framework to prevent the password leaking, and promptly discards the password after connecting to its database.

Access to data *via* the server's web front end is governed by user-based and group-based permissions. *Users* themselves may have superuser status (which gives unrestricted access to data and administrative functions via the front end), or be “locked” to a single patient record (when that user belongs to a patient/subject for “single-patient” mode), or be a routine “staff” user. Users may be a member of one or more groups. At any time, one group is selected to receive data uploaded by that user. *Groups* were discussed above. Groups define patient/subject identification criteria (e.g., fully identifiable vs. pseudonymised) and intellectual property restrictions. Groups “own” subsets of data, but groups (and thus their members) can also be granted permission to view data from specific other groups. *User–group associations (group memberships)* are associated with a further set of permissions: to administer the group (e.g., manage users within that group), plus individual permissions to upload data, to register new client devices, to log in *via* the web front end, to view data for multiple subjects when no subject search criteria have been applied, to export data in bulk, to run reports, or to attach notes to uploaded tasks. These permissions provide fine-grained control over what users can see and do, but a security breach of a group administrator account, or even worse a superuser account, would permit large-scale access to CamCOPS data held on the server.

The server must also be secured in other ways that are outside the scope of the CamCOPS system itself but are nevertheless critical. Standard security considerations include limiting physical access to the server; preventing visibility on public networks (e.g., limiting visibility to internal institutional networks or *via* secure VPN access to them); configuring a firewall appropriately; limiting secure shell (SSH) access; ensuring that the web server does not offer CamCOPS data by any route other than via the CamCOPS web front end itself; ensuring that no inappropriate users have access to the back-end database systems stored on the server; ensuring that the server is backed up regularly; ensuring physical security of backups; and ensuring server availability (e.g., in the face of power failure) should this be required.

### “Analytics” Security

It is commonplace amongst mobile applications to send information about application usage back to the application's creators. CamCOPS does not do this. No information is sent by the client app except to the chosen institutional server, and no information is exported by the server except as permitted or configured by the local administrator.

### Black Hat's Options

It is important to ask of any potentially sensitive system: what would it take to steal its data? Several methods are possible for CamCOPS:

- *Steal a device, the device's OS password, and its CamCOPS app password together*. This would allow existing records, still on that device, to be viewed.
- *Steal a device, the device's OS password, its CamCOPS app password, and its CamCOPS privileged-mode password together*. This would allow records still on that device to be sent to a “dark” server of the attacker's choosing.
- *Steal a user's CamCOPS server password, and a means of accessing the network on which the server is held*. This would allow the attacker to view data on the server (subject to the permissions granted to that user). If the server is on the open Internet, the network security requirement is eliminated, emphasizing the importance of network security for sensitive data, as well as strong passwords. This is the route of attack requiring particular security focus, since a predominant route of data theft is *via* “social engineering” rather than technical methods (76, 77). This risk is mitigated by requiring multi-factor authentication (as above).
- *Break into the server and gain direct access to its database*. This emphasizes the importance of securing the server.

These methods of attack may appear plausible but should not be possible:

- *Steal a device and the device's OS password, “root” the device to bypass factory default access restrictions, and access the tablet's CamCOPS SQLite database directly*. This would yield only CamCOPS app databases encrypted with AES-256.
- *Steal a tablet that has not been properly secured with a device (OS) password, or in other ways bypass the OS security*. As before, without the CamCOPS password, this would yield only an AES-256-encrypted database.
- *Steal a tablet and the tablet's OS password, download the open-source CamCOPS app, modify it, install it over the existing app without deleting the app data (bypassing any OS-specific digital signature checks on software installation), and attempt to use the modified app to export data*. Since the CamCOPS app does not know the password used to encrypt a given user's data, this conveys no benefit to the attacker; the database remains encrypted.

Once a computer is stolen, it can be dismantled. One must therefore consider also the possibility of breaking the encryption. No practical method is known of breaking the AES algorithm used to encrypt tablet data. The US National Security Agency approves AES for US government information classified Secret (for AES-128 or higher) or Top Secret (for AES-192 or higher) (67) and the UK NHS approves it for clinical data (16). CamCOPS uses AES-256. To give a sense of scale, a brute-force attack on an *n*-bit key takes a mean of 0.5 × 2^*n*^ + 0.5 cycles; therefore, a 256-bit key would take approximately 1.83 × 10^59^ years to discover by this method with a 10 GHz attack frequency. The universe is 1.38 × 10^10^ years old.

### Other Means of Ensuring Security of Patient-Identifiable Data

If a mobile device or other computer can “see” data on a remote server, then those data can be captured, even if by the simple expedient of saving a screenshot or taking a photograph of the device. This applies to any computer program, not just CamCOPS. Therefore, technical constraints are insufficient: users must be prohibited by cultural (institutional and/or legal) constraints from saving or storing patient-identifiable information on mobile devices in non-permitted ways. Similarly, users must be encouraged to look after their computer devices carefully, locking them when not in active use.

### Audit Trails

Client-side audit trails are minimal, but the app time-stamps all tasks at their creation, and time-stamps the last modification to any record, as well as collecting information relevant to the time it takes to complete each task. In contrast, there is significant audit logging on the server. The CamCOPS server maintains a number of task-specific tables. To each record, the server adds fields allowing an audit trail. When a record is modified or deleted, the old versions are kept. The server's tables therefore contain a snapshot of each device's current state, and a complete audit trail, whose granularity is the frequency of uploads from a particular device. Access requests to the server *via* the web interface are also audited and logged, as are command-line CamCOPS operations by administrators.

### Security Against Data Loss

Crashes in the CamCOPS app should not (and in our experience during development, do not) affect data integrity, because the SQLite back-end, with perhaps 500 million deployments worldwide (78), is designed to cope with this (79, 80). Additionally, only a small quantity of data is ever stored on the device, since data is regularly moved to the server, so the vulnerability to data loss from a device or app fault is in any case small. When the app upload its data, the process is atomic, meaning that the transaction either succeeds as a whole or fails as a whole, and does not leave the databases in a “halfway” state. Data on the server is typically stored using the well-established MySQL/MariaDB database system (48, 49).

Data loss remains possible. Reasons for this may include factors outside the CamCOPS system, such as a server environment that is insufficiently robust to cope with power loss or disaster. An amateurish example would be a server without an uninterruptible power supply (UPS). An example of server failure in an NHS high-availability environment was the Buncefield oil depot explosion on 11 December 2005, which temporarily disabled some laboratory computer systems used by our local acute hospital because a major computing provider was located near that depot.

As with any software system (81), it is also possible that the CamCOPS system might contain undiscovered bugs and therefore lose data. During development, in addition to human testing, several other steps are taken to minimize this possibility. CamCOPS includes an automated unit testing framework. We use a continuous integration (CI) service to run the automated tests every time the server code is changed, thus checking for software regressions, and the CI service also checks against a database of any reported security vulnerabilities in the Python packages used. For the client app, C++ compilation automatically detects some categories of error (82). We have a process of peer review for substantial code changes. In day-to-day operation, the server verifies that task information is complete, and valid (i.e., that all field values are permitted for that task), or warns the user accordingly. It also catches any potential internal errors to ensure that all transactions end in a database commit or a database rollback, meaning that any crashes that might occur within the server do not corrupt data or leave database locks held and block other processes.

However, CamCOPS is not presently accredited to NHS Interoperability Toolkit (ITK) standards or certified as a primary part of a clinical record. Therefore, a core requirement of data security would be to ensure that any information of sufficient importance be copied (e.g., in fully structured or PDF format) promptly from CamCOPS to a certified information storage system, such as an institution's primary EHR. To enable automatic copying of CamCOPS data into a certified information storage system, CamCOPS provides automatic export facilities (as above).

### Security and Risk Comparate

One matter that is easily overlooked in discussions of technical security measures is the *relative* security or risk of an electronic approach compared to its alternatives, which are often far from risk-free. In areas with no Internet connectivity, the alternative to storing patient-identifiable data on a mobile device is usually to write it down. Paper-based methods can be less secure than their electronic equivalents (83). In addition, manual scoring of cognitive assessment scales is vulnerable to assessor cognitive error (84–86) and this in itself represents a degree of clinical risk. Paper-based methods can also limit clinical information transfer, if handwriting is unclear or becomes unclear through photocopying or faxing, or if the time required to copy or summarize information means that only a subset of information is transferred.

### Legacy Security

Legacy security refers to the possibility that changes in hardware or software render old data inaccessible or unusable, such as when software applications refuse to start after expiry of a licence period. The CamCOPS code is open source, so can be installed, modified, and used freely by anyone, and should only include tasks/questionnaires that are in the public domain or where permission exists to use the task in perpetuity. As a last resort there is a clear procedure should the legal position on a task ever change, allowing removal of disputed content but preservation of all data: namely to remove or replace disallowed text and/or media from the app's and the server's resource files, leaving the code intact. This would result in a stripped-down data capture task and the ability to display and manipulate old data, as described above. Third-party code and development tools used by CamCOPS are similarly open source.

## Additional Considerations for Use in Clinical Practice and Research

### Software Regulations and Limitations

While we have tried to ensure that CamCOPS is reliable and accurate, the terms and conditions of use include a disclaimer to the effect that the authors and distributors are not responsible for errors or liable for any consequences of users' reliance upon the content provided with CamCOPS. Content contained in or accessed through CamCOPS should not be relied upon for medical purposes in any way; if medical advice is required, users should seek expert medical assistance. CamCOPS is intended for use under the supervision of medical practitioners or researchers conducting ethically approved academic research.

Regarding the European Union Medical Devices Directive (87): CamCOPS is not intended primarily for the diagnosis and/or monitoring of human disease. It has not yet undergone a conformity assessment under the Medical Devices Directive, and thus cannot be described as or put into service as a medical device. We note that Medical Device approval is typically not required for research software tools, during research where there is no medical purpose for the device (88); such research has its own regulatory controls. Nor is it typically needed for software systems where the software does not interpret data, merely storing and transmitting it without change (for example, Medical Device approval is not needed for word processors, spreadsheets, databases, or e-mail systems that may sometimes contain medical data) (89); many CamCOPS tasks relating to clinical work perform no such interpretation. We are continuing to explore this evolving area of regulation.

### Local Clinical and Research Approvals

In addition to these caveats, use within NHS England would require appropriate local NHS Trust approval (17). The CamCOPS system stores small quantities of patient-identifiable data on an encrypted mobile device for a limited period of time. NHS England guidelines allow this possibility subject to (a) strict rules regarding encryption, such as suitable cryptographic algorithms used with strong passwords; (b) all such devices being owned by the Trust, disallowing mobile devices owned by clinicians personally; and (c) Trust Information Governance and Caldicott Guardian approval (13, 16, 90). Device encryption on iPads uses AES-256 (71), while Android uses AES-128 (69, 91); both satisfy NHS encryption guidelines (16). CamCOPS data encryption, as above, is in addition to this. NHS Scotland guidelines classify data using a traffic-light system according to the risk of patient identification and harm or distress caused by loss (92). Patient-identifiable data relating to mental states would be classified as amber or red—likely often red. When applied to the CamCOPS system, which holds information transiently offline on a mobile device, these standards would require NHS-owned devices with whole-disk encryption and a strong password (92).

As noted above, CamCOPS is not a primary EHR system and it is critical that any clinically relevant data be copied to an institution's primary EHR. CamCOPS provides mechanisms for this to occur automatically (see above), subject to the EHR having the capability to receive it (see below for discussion of one possible fallback position with EHRs that do not).

In a research context, information-handling procedures will be directed by an appropriate national or institutional research governance framework [e.g., (93, 94)]. Clinical information governance guidelines are typically at least as stringent as guidelines that govern research with volunteers who have given explicit consent to research, and more stringent than guidelines covering pseudonymised or anonymised records, or non-sensitive information. CamCOPS was therefore designed against clinical information governance standards.

All tasks allowing free-text entry, and many established structured questionnaires in psychiatry, permit the capture of risk-related information, such as about suicidality. If such information is captured without direct supervision by a clinician, it is vital that a clinical service or research study has approved methods for handling such information. Most critically, patients/subjects must be aware that reporting information to an app is not a substitute for talking to their clinical/research team or obtaining emergency health care if required. Users must indicate that they understand this in order to use the app, but appropriate expectations must also be set by the institution operating the software.

### Experimental Tasks

CamCOPS is designed to operate as a translational research platform, implementing human-specific and cross-species tasks derived from basic neuroscience research. Some experimental tasks are included in CamCOPS and are clearly labelled as such; more may be added.

## Early Experiences

CamCOPS development began in 2012 and the first version of the client, written in the Titanium cross-platform Javascript framework (95), was available in 2013 together with a Python-based server. The system was developed incrementally, except that in 2017 the client was rewritten in C++/Qt for better performance and power, and the server reworked. CamCOPS was first approved for clinical use in October 2014 within Cambridgeshire & Peterborough NHS Foundation Trust (CPFT), and has been used both for clinical and research purposes. It has been deployed for research within CPFT, the University of Cambridge, and at academic institutions in Denmark and Singapore. It has been used on Android tablets including the Asus TF201, Asus TF300T, and Sony Xperia Z2 Tablet, and touchscreen Windows devices including the Microsoft Surface Book 2. Our experience has been that Windows tablets provide familiarity and multi-purpose computing for many users, whilst Android tablets can be cheap. All these operating systems support offline voice-recognition dictation systems, as described above, though we have found physical keyboards considerably more accurate for text entry.

As a clinical example, CamCOPS has been operational in CPFT's perinatal mental health service since 2019, where it is used to record questionnaire data relating to symptoms and service experience. Notably, the version of the EHR system in use did not have the capability to receive an automatic data “feed” from CamCOPS. We therefore used the poor substitute of having CamCOPS automatically e-mail tasks (on receipt) *via* an internal secure e-mail system to an administrative team, who uploaded them to the patient's EHR.

Examples in a research context include the Insight study (96) and MOJO study (Khandaker, NHS research ethics reference 19/EE/0233) examining the relationship between systemic inflammation and mood symptoms, in which CamCOPS has been used to capture a range of data encompassing medical history, affective symptoms including a standardized self-report computerized interview (21, 22), physical symptoms such as fatigue and joint inflammation, and quality-of-life measures.

## Comparison to Other Systems; Strengths and Weaknesses

There are a large number of free and commercial applications offering data capture for psychiatry-oriented questionnaires such as the PHQ-9, and similarly many web sites for users to design and offer generic surveys for free or *via* a variety of commercial models. Some systems offer extensively validated complex cognitive assessment tasks *via* a closed-source model with provider-hosted data [e.g., (97, 98)]. Others use a range of data collection techniques (mobile apps, web interfaces, text messaging) to collect information in specific clinical domains, such as for mood monitoring [e.g., (99)] or to detect psychiatric morbidity in general hospital contexts [e.g., (100)]. REDCap (31) is one widely used general-purpose research system, free but not open source (101–103), oriented towards flexible online data capture and using a model where institutions host their own instance (31, 102, 103).

CamCOPS differs from these systems in some ways, and at times complements them. Of course, all major design decisions come with trade-offs. We see the major decisions as follows.

Firstly, CamCOPS is free and open-source software; moreover, it has a “copyleft” licence that ensures derivative works must remain open source. This eliminates direct software costs and allows public scrutiny of the code, but may reduce the incentive for commercialization and commercial support. It also prevents the full incorporation of tasks incompatible with this licensing model. Careful intellectual property review is required with respect to new tasks (see above), though that would be true regardless of the software licence.

Second, we follow the principle of institutional hosting. This offers institutions complete ownership and control of their data, but comes with the burden of having to provide, obtain, or outsource relevant computing infrastructure and some burden of computer administration.

Third, CamCOPS can operate offline. This major design decision reflected our need to operate in offline environments such as on domiciliary visits to mobile phone (cellular data) “black spots” for our network providers, or in acute hospital environments with radiofrequency shielding or lack of Wi-Fi for other reasons. This inevitably excludes the much simpler software model where all testing is performed online *via* a web site, and it brings complexities in development, data security management (discussed above), and deployment (such as upgrading client apps). A benefit is that the client, being written in a high-performance low-level general-purpose programming language, is essentially unrestricted; thus, CamCOPS can and does implement animated tasks, generalized linear modelling, and so forth.

Fourth, we support on-device “registration” of new subjects/patients, and support multiple groups and identification policies. This adds clinical flexibility (e.g., capturing data in relation to an emergency referral prior to administrative patient registration) and supports a variety of clinical and research settings, from fully identified clinical work, to a mix of clinical and research work, to de-identified research. However, it adds complexity and can require more later verification than a model in which all patients are registered in advance on the server according to a unified identity policy. In practice, since the identity policy (or policies) is configured by the local system administrator, this balance is in large part determined by the hosting institution according to its needs.

Fifth, the tight security for data stored transiently on mobile devices, with its principle of data minimization, brings some trade-offs, such as the absence of a view of historical data “on the fly” within the mobile app. If historical CamCOPS information needs to be viewed, that is presently not supported “offline” but only *via* online web access to the server. This may limit utility in some situations.

Sixth, tasks are implemented at present as part of the CamCOPS code base, rather than being user-defined [cf. e.g., (31)]. An advantage is that tasks are developed as “canonical” versions, with their source code open—for example, everyone can check to see if there is a logical error in the implementation of a task. We have also found that the requirement to implement aspects of each task in both C++ and Python serves as an intrinsic cross-check for this kind of error, although it involves some extra work. It also brings the benefit that tasks are unconstrained—that is, they can use any feature of a general-purpose programming language—rather than being constrained by the limitations of a scripting environment, so they can be tailored to achieve a good user interface and experience. The obvious disadvantage is that CamCOPS is not well-suited for the creation of new questionnaires specific to a clinical service or research study on a rapid, *ad hoc* basis (including research workflow tasks such as recording consent); CamCOPS may therefore complement software designed for that purpose in a clinical [e.g., (104)] or research [e.g., (31)] environment. It also requires more programming experience to develop new tasks than simpler systems.

Finally, we note that in the clinical domain there is often tension between different modes of data capture that we see as stemming from a lack of interoperability. Many EHR systems are not designed to be used by patients at all, but are designed for clinicians to enter data. In the UK, this is changing gradually with the advent of “patient portals” and EHR-connected apps, but while some EHRs can capture basic questionnaire data from patients directly, we know of none that can capture structured data from complex clinician-assisted tasks [e.g., (3)] or animated cognitive assessments [e.g., (23)] directly into the EHR *via* a convenient interface. This creates demand for systems that can, and that situation is likely to persist—primary EHR systems do a lot, but they cannot do everything. Accordingly, we suggest that the future focus in this area should be on using the “best tool for the job”—capturing directly into the EHR as the first preference, but using external tools (such as CamCOPS or others) where required—plus work to improve the integration of external systems and EHRs, so that data flows seamlessly in the most structured way possible as well as the most clinically relevant.

## Summary

Regardless of the current and future sophistication of phenotype measurement *via* passive data collection (105), in our view overt data capture will continue to remain central to digital phenotyping in psychiatry. We present CamCOPS, a free and open-source client–server system for direct data capture in the general area of psychiatry, psychology, and the clinical neurosciences. It runs on multiple platforms and emphasizes touchscreen data capture. It has both clinical and research applications and is designed to operate against stringent information governance requirements, with hosting institutions having complete ownership and control of the data they collect. It can operate with fully identifiable or de-identified information. We discuss security concerns that would apply to any system of this kind, and describe the approaches used in CamCOPS. It provides summary views on the data that we believe are useful for clinicians, whilst retaining full structured data for research, and it supports multiple export mechanisms to communicate with other systems. It implements a large and growing family of tasks, ranging from questionnaires to animated cognitive assessments, with techniques to address a range of licensing and intellectual property rules. We discuss its strengths and weaknesses and report on some early practical uses.

## Data Availability Statement

Publicly available datasets were analyzed in this study. This data can be found at: https://camcops.readthedocs.io/; https://github.com/RudolfCardinal/camcops.

## Author Contributions

RC designed and wrote CamCOPS (2012−) and drafted the manuscript. MB contributed to the design and development (2019−). Both authors contributed, edited, and approved the final manuscript.

## Funding

RC was supported by a Wellcome Trust postdoctoral fellowship (091998/Z/10/Z). RC's and MB's research was supported by a UK Medical Research Council (MRC) Mental Health Data Pathfinder grant (MC_PC_17213 to RC). Deployment was supported in part by the UK National Institute of Health Research (NIHR) Cambridge Biomedical Research Centre (BRC-1215-20014). The work was conducted within the Behavioural and Clinical Neuroscience Institute, supported by the Wellcome Trust (093875/Z/10/Z) and the MRC (G1000183).

## Author Disclaimer

The views expressed are those of the author and not necessarily those of the NHS, the NIHR, or the Department of Health and Social Care.

## Conflict of Interest

RC consults for Campden Instruments Ltd., in the area of research software and receives royalties from Cambridge University Press, Cambridge Enterprise, and Routledge. The remaining author declares that the research was conducted in the absence of any commercial or financial relationships that could be construed as a potential conflict of interest.

## Publisher's Note

All claims expressed in this article are solely those of the authors and do not necessarily represent those of their affiliated organizations, or those of the publisher, the editors and the reviewers. Any product that may be evaluated in this article, or claim that may be made by its manufacturer, is not guaranteed or endorsed by the publisher.
